# NIA DIVISION OF GERIATRICS AND CLINICAL GERONTOLOGY

**DOI:** 10.1093/geroni/igae098.0331

**Published:** 2024-12-31

**Authors:** Basil Eldadah

**Affiliations:** National Institute on Aging, Baltimore, Maryland, United States

## Abstract

Dr. Eldadah will discuss research priorities of the NIA Division of Geriatrics and Clinical Gerontology. Dr. Eldadah will also be available for small group discussion.

